# Cancer: An Oxidative Crosstalk between Solid Tumor Cells and Cancer Associated Fibroblasts

**DOI:** 10.1155/2016/4502846

**Published:** 2016-08-09

**Authors:** Alessandro Arcucci, Maria Rosaria Ruocco, Giuseppina Granato, Anna Maria Sacco, Stefania Montagnani

**Affiliations:** ^1^Department of Public Health, University of Naples Federico II, 80131 Naples, Italy; ^2^Department of Molecular Medicine and Medical Biotechnology, University of Naples Federico II, 80131 Naples, Italy

## Abstract

Redox balance is associated with the regulation of several cell signalling pathways and functions. In fact, under physiological conditions, cells maintain a balance between oxidant and antioxidant systems, and reactive oxygen species (ROS) can act as second messengers to regulate cell proliferation, cell death, and other physiological processes. Cancer tissues usually contain higher levels of ROS than normal tissues, and this ROS overproduction is associated with tumor development. Neoplastic tissues are very heterogeneous systems, composed of tumor cells and microenvironment that has a critical role in tumor progression. Cancer associated fibroblasts (CAFs) represent the main cell type of tumor microenvironment, and they contribute to tumor growth by undergoing an irreversible activation process. It is known that ROS can be transferred from cancer cells to fibroblasts. In particular, ROS affect the behaviour of CAFs by promoting the conversion of fibroblasts to myofibroblasts that support tumor progression and dissemination. Furthermore, the wrecking of redox homeostasis in cancer cells and tumor microenvironment induces a metabolic reprogramming in tumor cells and cancer associated fibroblasts, giving advantage to cancer growth. This review describes the role of ROS in tumor growth, by focusing on CAFs activation and metabolic interactions between cancer cells and stromal fibroblasts.

## 1. Introduction

**Figure 1:**
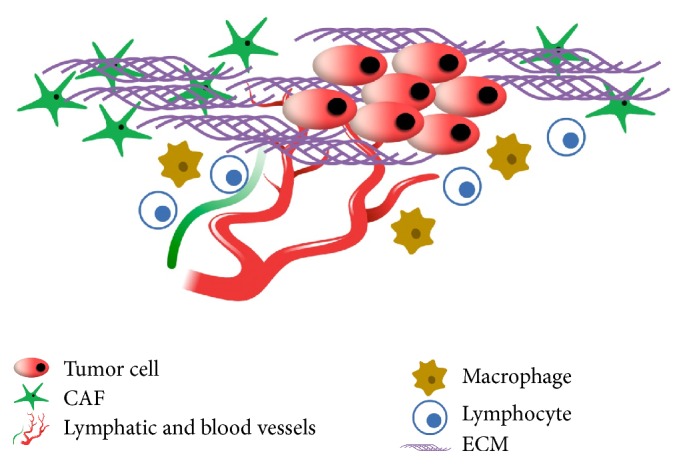
Typical components of solid tumors microenvironment.

Solid tumors are characterized by an abnormal microenvironment regulating tumor progression and providing evasion from cancer therapies [1]. Cancer microenvironment includes blood and lymphatic tumor vessels, extracellular matrix (ECM), and noncancer stromal cells [2] (Figure 1). Blood and lymphatic vessels have a significant role in cancer growth and metastasis [3, 4]. ECM is formed by interstitial matrix and basement membrane and is very important for the anchorage and migration of cancer cells [2]. Furthermore, ECM contains and stores soluble molecules that can become available for tumor cells. These molecules include chemokines such as CXCL-12, matrix metalloproteinases (MMPs), protease inhibitors, and growth factors such as vascular endothelial growth factor (VEGF) [2]. Noncancer stromal cells are represented by endothelial cells, pericytes, immune cells, cancer associated fibroblasts (CAFs), activated adipocytes, and mesenchymal stem cells (MSCs) [1]. Moreover, all components of tumor microenvironment may vary, depending on the type and location of tumor, thus making each tumor unique [1]. Unlike normal fibroblasts that regulate the turnover of the ECM, control normal tissue homeostasis, and participate in wound healing and senescence, CAFs either reside within the tumor margins or infiltrate the tumor mass and facilitate the tumorigenesis [5]. In particular, the phenotype of CAFs is different from that of normal fibroblasts and is characterized by a more rapid proliferation rate, enhanced collagen production, secretion of growth factors, and ECM modulators [5]. Noncancer stromal cells of almost all solid tumors contain CAFs, whose abundance varies between different cancer types. In particular, breast, prostate, and pancreatic cancers contain high numbers of CAFs; conversely, a reduced number of CAFs is usually present in brain and renal cancers [6]. In breast and pancreatic carcinoma CAFs can represent up to 80% of the tumor mass, as a result of a widespread desmoplasia [7]. Cancer tissues containing a high percentage (>50%) of CAFs present increased microvessel density, tumor-associated macrophages, epithelial-mesenchymal transition (EMT), and poor prognosis [7]. CAFs include two cell populations, such as fibroblasts and myofibroblasts, and are represented by nonneoplastic, nonvascular, nonepithelial cells with a fibroblastic phenotype [6]. Several markers are used to detect CAFs in cancer tissues; however, *α*-SMA is the most widely used marker, because there are more myofibroblasts in tumor stroma and *α*-SMA is a specific marker for myofibroblasts [8–10].

Moreover, myofibroblasts represent a cell population of cancer desmoplastic tissues. Indeed, a histological hallmark of cancer tissue is represented by the formation of an abundant tumor stroma formed by ECM and myofibroblasts, inflammatory cells, blood, and lymphatic vessels: this process, named desmoplasia, wrecks the organ's normal architecture [11]. Desmoplasia creates a niche for cancer cells, represents a sign of tumor progression, and can be used as intraoperative prognostic marker [12–14]. In particular, desmoplasia generates mechanical forces that convert fibroblasts and other precursor to myofibroblasts, and it originates a hypoxic and acidic microenvironment that compromises chemotherapeutic treatment [15]. Moreover, hypoxic microenvironment of desmoplastic cancer tissues produces and maintains an oxidative stress condition, because hypoxia is associated with mitochondrial ROS production and glycolytic pathway [16].

However, the whole cancer tissue contains high levels of ROS. In particular, both cancer cells and CAFs produce reactive oxygen species (ROS), such as hydrogen peroxide (H_2_O_2_) that induces myofibroblast phenotype in CAFs and cancer alterations in epithelial cells: these processes influence the behaviour of neighboring CAFs and cancer cells [16, 17].

Therefore, this review describes the role of ROS in CAFs activation and in the regulation of tumor-stroma interaction.

## 2. ROS in Cancer Cells

ROS are a group of chemical species, unstable and reactive derivatives of oxygen. They are represented by electrophilic and highly reactive molecules such as radical forms represented mainly by superoxide anion (O_2_^∙−^), hydroxyl (OH^•^), carbon dioxide anion (CO_2_^∙−^), and nonradical forms, such as H_2_O_2_ [16]. ROS sources can be endogenous or exogenous [18]. Endogenous ROS are produced mainly by mitochondria during oxidative phosphorylation (OXPHOS) process, NADPH oxidase enzymes (NOX), and cytochromes: the ROS production begins with the transfer of one electron to molecular oxygen (O_2_) to form O_2_^∙−^ which can dismutate into H_2_O_2_ spontaneously at low pH or through enzymatic activity of the superoxide dismutase (SOD) enzyme [16, 19]. Moreover, H_2_O_2_ can be transformed, through the Fenton reaction, to highly reactive and toxic OH^*∙*^ [16, 19]. Exogenous ROS sources are represented by pollutants, tobacco, smoke, drugs, xenobiotics, and radiations [18, 20].

ROS homeostasis depends on the balance between ROS production and enzymatic and nonenzymatic antioxidant systems; indeed, the wrecking of this equilibrium leads to oxidative stress, which can contribute to tumor development [21]. In particular, oxidative stress can alter the cellular physiological functioning of the redox-sensitive mechanisms, without inducing an irreversible oxidative damage or general oxidative cell condition [21]. Moreover, the typical hypoxic tumor microenvironment regulates cancer invasiveness through mitochondrial redox signalling [22]. Hypoxia induces the mitochondrial production of O_2_^∙−^ that is transformed to H_2_O_2_ by SOD2 enzymatic activity [21]. H_2_O_2_, one of the most stable and permeable ROS, can traverse the mitochondrial and cell membranes and it represents the main ROS in the regulation of signalling transduction pathways, affecting the behaviour of cancer and stromal cells [16, 19, 22]. Furthermore, aquaporin-8 that is a transmembrane protein localized in the plasma and inner mitochondrial membrane facilitates the diffusion of H_2_O_2_ across cellular membranes [23]. In a hepatocellular carcinoma cell line the silencing of* aquaporin-8* gene impairs H_2_O_2_ mitochondrial releases, increases mitochondrial ROS, and induces a ROS-dependent mitochondrial depolarization as well as a loss of cell viability [24]. Moreover, there are several works showing and explaining the positive function of aquaporin-8 in carcinogenesis [25–28]. In conclusion, the perturbation of cellular redox homeostasis can represent one of the main factors inducing and maintaining tumor phenotype in cancer tissues.

## 3. ROS and Cancer Associated Fibroblasts Activation

The pivotal role of microenvironment in tumor development, progression, and metastasis is known. In particular, the most abundant cell population in the stroma of solid tumors is represented by CAFs [29]. CAFs play a key role in tumorigenesis, tumor support, and progression and are characterized by a heterogeneous cell origin. Although CAFs can derive from epithelial or endothelial cells through an epithelial or endothelial mesenchymal transition (EMT/EndMT) [30–32], EMT/EndMT cannot be considered the only origin of CAFs. CAFs can also derive from hematopoietic stem cells [33], pericytes, and adipocytes [34], although tumor stroma resident fibroblasts are one of the main sources of CAFs [35, 36]. However, most of CAFs are phenotypically and epigenetically different with respect to normal dermal fibroblasts. Indeed, CAFs can be considered a cell population in a permanent activated state, sharing similarities with fibroblasts activated during wound healing [37]. The activated CAFs promote tumorigenesis and cancer development through secretion of growth factors, cytokines, and paracrine interactions [6]. In breast cancer over 80% of CAFs are myofibroblasts that, contrary to wound healing process, are constitutively activated and neither revert to a normal phenotype nor undergo apoptosis [38]. Breast CAFs show high expression of *α*-SMA, p53, podoplanin, CD10, fibroblast activation protein (FAP), matrix metalloproteinases (MMPs), tenascin-C, and platelet-derived growth factor receptor (PDGFR*α*/*β*) and lose caveolin-1 (Cav-1) expression [39]. Other markers such as fibroblast-specific protein 1 (FSP1), neuron-glial antigen 2 (NG2), and prolyl 4-hydroxylase can be useful to identify CAFs subtypes in carcinomas [6]. The irreversible activation of CAFs is due to signalling pathways driven by several factors produced mainly by tumor cells and autocrine loops [16, 37]. Different studies showed that ROS have a pivotal role in the process of fibroblast activation. TGF-*β*1, involved in fibroblast-to-myofibroblast switch, induces an increase of fibroblasts ROS that mediate the expression of *α*-SMA [40]. One of the main sources of this ROS boost seems to be represented by NOX associated with fibroblasts membranes [41, 42], and in particular many studies have observed that NOX4 represents the main target of TGF-*β*1 in the process of fibroblasts activation [43–45]. Furthermore, TGF-*β*1 activates a mitochondrial ROS production that is also required for* Nox4* transcription. Hence, NOX4 expression, amplifying the ROS mitochondrial burst, can prolong TGF-*β*1 signalling [46]. In addition, the unregulated mitochondrial ROS production is associated with fibrosis evolution as well as cancer progression and invasion [16, 47]. In fibroblasts, a genetic dysfunction of mitochondrial complex I produces an oxidative stress triggering fibroblasts activation; under these conditions, myofibroblasts promote a proinvasive phenotype of human melanoma cells through the secretion of cytokines [48]. Oxidative status of tumor stroma can affect cancer cells dissemination. Indeed, it has been observed that, in a model of mammary carcinogenesis,* junD* ^−/−^ stromal fibroblasts increase the metastatic potential of neoplastic cells [49]. JunD, a member of activator protein-1, positively regulates some antioxidant genes [50]. Furthermore in a* junD* ^−/−^ fibroblasts system the activation of fibroblasts into myofibroblasts was linked to an increase of oxidative status [49]. Conversely, the decrease of oxidative stress in tumor stroma can reduce cancer mass and revert its metastatic potential. Indeed, it has been shown that Cav-1 and SOD2 in CAFs can act as oncosuppressors [51]. Moreover, in prostatic hyperplasia and prostate cancer, the upregulation of antioxidant selenoenzymes, such as glutathione peroxidase 3 and thioredoxin reductase I, inhibits the differentiation of fibroblasts into myofibroblasts through the reduction of ROS levels produced by NOX4 [52].

ROS can affect the differentiation and the tumorigenic potential of a heterogeneous cell population, such as CAFs, through different mechanisms. Indeed, in a PDGFR-*β* fibroblast subtype ROS mediate PDGF signalling through phosphatases inactivation [53]. It has been shown that the reversible inhibition of phosphatases by PDGF-induced ROS is involved in the fibroblast proliferation and migration [54]. Many studies have evidenced NOX as the main source of ROS induced upon growth factors signalling [55–57], although also mitochondrial ROS can contribute to the oxidation and inhibition of protein phosphatases [58]. Furthermore, ROS modulated Cav-1 expression is closely linked to the role of CAFs as tumor feeder and to biochemical remodelling of the microenvironment [16]. Hence, it is possible that different ROS species can evoke specific effects on Cav-1^+^ CAFs. Indeed, it has been demonstrated that in lung cancer cells Cav-1 is differentially modulated by various ROS [59]. In the complex, ROS represent main factors in the fibroblast activation process as well as in CAFs behaviour, although further studies will be necessary to better understand signalling pathways activated by ROS.

## 4. ROS and Metabolic Interaction between Stromal Fibroblasts and Cancer Cells

It is known that cancer tissues produce high levels of ROS that can derive from mitochondrial dysfunction, upregulation of NOX1 and NOX4, and alterations of antioxidant enzymes [16]. In particular, the mitochondrial dysfunction due to impairment of mitochondrial electron transport chain produces a considerable O_2_^∙−^ production [16]. The mitochondrial dysfunction is usually associated with a switch to aerobic glycolysis, known as Warburg effect that is an early step of carcinogenesis, and can occur before the appearance of a hypoxic tumor environment [60]. In fact, lung cancer cells grow in the presence of oxygen and utilize glycolysis to produce ATP [60]. Glycolysis produces lower amount of ATP with respect to OXPHOS, but the glycolytic choice offers to cancer cells a fast ATP production and glycolytic intermediates that contribute to the growth of cancer cells [19, 60]. During glycolysis the transformation of pyruvate into lactate leads to a deficit of pyruvate, the primary fuel supporting Krebs cycle: this process deprives cancer cells of some Krebs cycle intermediates with antioxidant activity [16]. However, hypoxia is the main factor inducing glycolytic switch in cancer cells: it is associated with ROS production by mitochondrial complex III and induces the activation of HIF-1*α* that contributes to glycolytic pathway [61]. In particular, HIF-1*α* regulates the switch from OXPHOS to glycolysis through the induction of enzymes involved in the glycolysis and overexpression of glucose transporters GLUT1 and GLUT3 [62]. Moreover, HIF-1*α* can be activated also by PI3K/AKT/mTOR signalling pathway, and HIF-1*α* levels can be correlated with the severity of many tumors [60, 63]. Therefore, in cancer cells both Warburg effect and mitochondrial malfunctioning induce an increase of lactate and ROS levels and a decrease of antioxidant molecules [16].

It is known that cancer cells produce large amounts of H_2_O_2_ without exogenous stimulation [64]. H_2_O_2_ produced and secreted by cancer cells “fertilizes” the tumor microenvironment and induces oxidative stress in CAFs. Martinez-Outschoorn studied H_2_O_2_ stromal effect, by coculturing a breast cancer cell line with immortalized fibroblasts [65, 66]. In particular, the transfer of oxidative stress from cancer cells to CAFs is associated with the reduction of mitochondrial function and the increase of both glucose uptake and ROS levels in CAFs [65]. On the other hand, cocultured cancer cells show significant increase of mitochondrial activity and decrease of both GLUT1 expression and glucose uptake. This process is interrupted if catalase is added to the cell culture media [65]. Furthermore, fibroblasts cocultured with breast cancer cells acquire a CAFs phenotype characterized by Cav-1 downregulation, increased expression of myofibroblast markers, extracellular matrix proteins, and constitutive activation of TGF-*β*/Smad2 signalling pathway [66]. In particular, TGF-*β* induces differentiation of prostate CAFs to myofibroblasts by triggering NOX4 upregulation and elevated ROS production [52]. Moreover, TGF-*β* triggers in fibroblasts increased oxidative stress, autophagy/mitophagy, aerobic glycolysis, and downregulation of Cav-1: these alterations can extend to surrounding fibroblasts and support cancer cell growth [67]. Loss of stromal Cav-1 may be used as biomarker for cancer aggressiveness [68, 69]. Cav-1 is a structural component of caveolae that are flask-shaped invaginations of the plasma membrane enriched in sphingolipids and cholesterol and are involved in several cellular functions, such as vesicular transport, cholesterol homeostasis, and signal transduction pathways [70, 71]. In particular, Cav-1 can regulate many transduction pathways by interacting with several signalling proteins localized in lipid rafts and caveolar membranes [72]. A recent work showed that Cav-1 is a negative regulator of ROS produced by NOX enzymes and this is realised by several mechanisms such as enzyme binding and inhibition of endogenous* Nox* gene expression [73]. In particular Cav-1 can inhibit ROS production from NOX2 and NOX5 by direct enzyme binding, via allosteric regulation. Moreover, in addition to this posttranslational regulation, Cav-1 represses NOX2 and NOX4 gene expression and protein synthesis through inhibition of the NF-*k*B pathway [73]. ROS produced by cancer cells induce loss of Cav-1 in stromal cells, driving glycolysis switch and lactate excretion. This process is associated with HIF-1*α* stabilization and upregulation of the monocarboxylate transporter MCT4, which is a biosensor of oxidative stress in CAFs [74, 75]. On the other hand, breast cancer cells, cocultured with fibroblasts, showed the upregulation of MCT1, the transporter involved in lactate uptake [74]. Hence, cancer cells induce in CAFs oxidative stress and a glycolysis switch associated with Cav-1 downregulation. In particular, loss of Cav-1 could be a marker of glycolysis in CAFs that increase in cancer cells OXPHOS rate, ATP production, and proliferation, by supplying tumor cells with lactate in a paracrine manner [76]. Furthermore, 3-hydroxybutyrate and L-lactate, end-products of aerobic glycolysis, feed tumor growth and metastasis in a human tumor xenograft experimental system [77]. In particular, 3-hydroxybutyrate induces an increase in tumor volume without any increase in angiogenesis, whereas L-lactate stimulates significantly the formation of metastases [77]. This paracrine metabolic crosstalk between CAFs and cancer cells could also represent a mechanism that confers drug-resistance during antiangiogenic therapy, by reducing the dependence of cancer cells on a vascular blood supply [78, 79]. Finally, it is important to note that standard chemotherapies induce in dermal fibroblast cell lines an activated CAFs phenotype, characterized by *α*-SMA expression, glycolytic switch, ROS production, senescence, autophagy, and increased secretion of interleukin 6. Hence, this catabolic and inflammatory microenvironment represents an ideal niche to sustain carcinogenesis [80].

Therefore, these observations lead to considering ROS as a bridge between tumor cells and CAFs, thus contributing to cancer development, progression, and metastasis.

## 5. Conclusions

Cancer tissues represent a network formed by cancer cells and microenvironment, where the close interactions between tumor cells and CAFs contribute to cancer growth and progression. Tumors show higher ROS levels than normal tissues, and ROS alterations have significant implications in tumor growth and metastasis. ROS induce tumorigenesis by affecting the behaviour of both tumor cells and CAFs and regulating their metabolic interactions. On the other hand, it is important to note that ROS are also involved in the activation of natural defences against the appearance and dissemination of cancer cells [20]. In particular, oxidative stress can limit distant metastasis of melanomas* in vivo* [81]. Moreover, successfully metastasizing melanomas undergo reversible metabolic changes, raising their capacity to resist oxidative stress. These metabolic changes include increased dependence upon NADPH-generating enzymes in the folate pathway, which is closely linked to GSH regeneration [81]. However, when ROS levels in tumor tissues become extremely high, they can induce cancer cell death. Indeed, the cytotoxicity of many chemotherapeutic drugs is mediated by a further increase of ROS levels in tumor tissues [82]. However, upon drug treatment some cancer cells can undergo a process of “redox resetting” associated with a new redox balance characterized by higher levels of ROS and stronger antioxidant systems. “Redox resetting” enables tumors to become resistant to anticancer drugs: it is possible to bypass this problem by combining drugs that generate ROS with compounds that downregulate the cellular antioxidant capacity [82]. Furthermore, recent works have demonstrated that CAFs not only play an important role in tumorigenesis but also contribute to chemotherapy resistance [83]. Hence, future therapeutic strategies will require the targeting of both cancer cells and stromal fibroblasts.
